# A Rare Pediatric Case of Severe Bird Fancier’s Lung Presented with Viral Pneumonitis-Like Picture

**DOI:** 10.3390/children5110149

**Published:** 2018-11-12

**Authors:** Basel Habra, Atqah AbdulWahab

**Affiliations:** 1 Department of Pediatric Medicine, Hamad Medical Corporation, Doha 3050, Qatar; atqah2015@gmail.com; 2 Sidra Medicine, Doha 26999, Qatar; 3 Weill Cornell Medicine, Doha 24144, Qatar

**Keywords:** Bird Fancier’s Lung (BFL), hypersensitivity pneumonitis (HP), chILD, pulse steroid

## Abstract

Bird Fancier’s Lung (BFL) is a rare, nonatopic immunologic response to repeated or intense inhalation of avian (bird) proteins/antigens found in the feathers or droppings of many species of birds, which leads to an immune-mediated inflammatory reaction in the respiratory system. Although this is the most common type of hypersensitivity pneumonitis (HP) reported in adults, it is one of the classifications of a rare subtype of interstitial lung disease that occurs in the pediatric age group of which few case reports are available in the literature. The pathophysiology of HP is complex; numerous organic and inorganic antigens can cause immune dysregulation, leading to an immune-related antigen–antibody response (immunoglobulin G—IgG- against the offending antigen). Diagnosing BFL in the pediatric age group is challenging due to the history of exposure usually being missed by health care providers, symptoms and clinical findings in such cases being nonspecific and often misdiagnosed during the acute illness with other common diseases such asthma or acute viral lower respiratory tract infection, and the lack of standardization of criteria for diagnosing such a condition or sensitive radiological or laboratory tests. Treatment, on the other hand, is also controversial. Avoidance of the offending antigen could be the sole or most important part of treatment, particularly in acute mild and moderate cases. Untreated cases can result in irreversible lung fibrosis. In this case report, we highlight how children presenting with an acute viral lower respiratory tract infection can overlap with the acute/subacute phase of HP. Early intervention with pulse steroids markedly improves the patient’s clinical course.

## 1. Case Report

**Figure 1:**
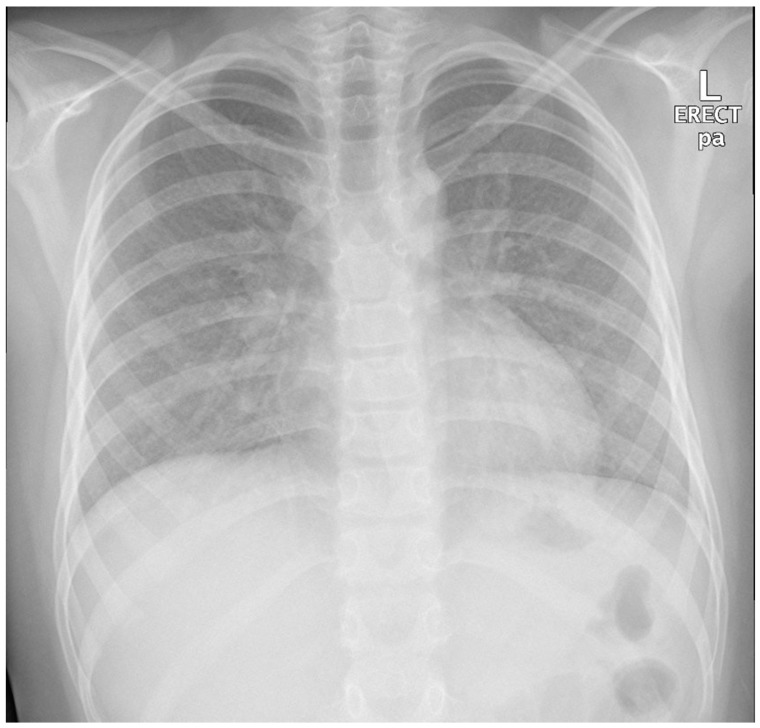
Patient first chest X-ray (CXR) showed bilateral diffuse miliary nodules.

**Figure 2:**
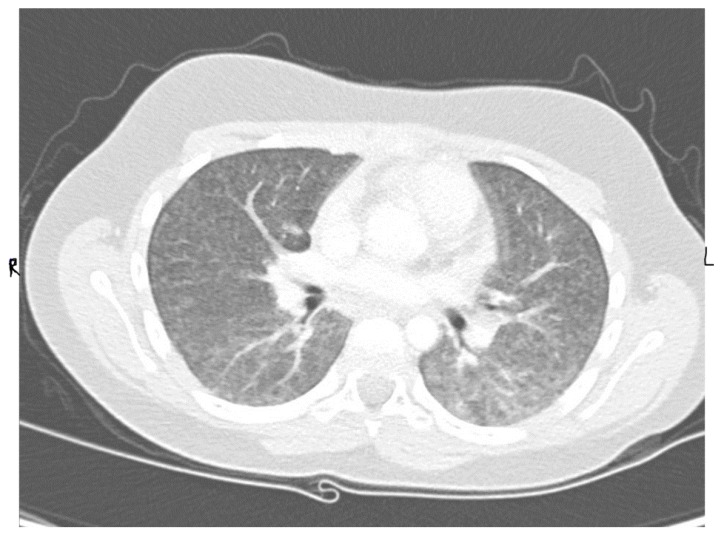
Patient CT scan of the chest showed diffusely reticular-nodular opacities in both lungs involving lung bases and the posterior segments of the right and left upper and lower lobes with irregular bronchovascular marking.

An 11-year-old, previously healthy boy presented to the emergency department with a 1-week history of dry cough, shortness of breath, and low-grade fever, with no associated symptoms of expectoration, hemoptysis, wheezing, contact with sick people, or recent travel, and no history of weight loss. His past medical history was unremarkable, and he was fully vaccinated in accordance with his age. Physical examination revealed an afebrile child with remarkable tachypnea with moderate respiratory distress, desaturation requiring oxygen, subcostal and intercostal retractions, and decreased air entry bilaterally on lung auscultation without wheeze or crackles. A chest X-ray (CXR) showed bilateral diffuse miliary nodules (Figure 1). The child required pediatric intensive care unit (PICU) admission for close observation and further management. He was started on IV cefuroxime and oral clarithromycin and Tamiflu as treatment of viral pneumonitis with possible secondary bacterial infection and required a high-flow nasal cannula (HFNC) for oxygen support. Respiratory virus polymerase chain reaction (PCR) showed bocavirus positive, at which point Tamiflu was discontinued. The child remained tachypneic with persistent respiratory distress and continued to require high oxygen therapy. Repetition of his CXR imaging showed persistent diffuse bilateral miliary infiltrations. A CT scan of the chest showed diffusely reticular-nodular opacities in both lungs involving lung bases and the posterior segments of the right and left upper and lower lobes with irregular bronchovascular marking (Figure 2). Purified protein derivative (PPD) and QuantiFERON-Gold showed negative results. The child showed improvement of his symptoms on day 10 of his hospital admission and was able to be weaned off oxygen support, and he was discharged in a stable general condition with a mild dry cough.

A few days after discharge, the child presented again to the emergency department with a worsening cough and progressive shortness of breath, also with physical findings of hypoxia and respiratory distress, and he required admission to our tertiary hospital. His physical examination revealed an afebrile child with tachypnea and hypoxia requiring 2 L of oxygen via nasal cannula, grade 1 digital clubbing, mild respiratory distress with nasal flaring, and decreased air entry bilaterally on lung auscultation without wheezing or crackles. His arterial blood gas showed a PH of 7.39, a PCO2 of 45 mmHg, a PO2 of 60 mmHg, and a BE of 2 and he was unable to perform a pulmonary function test (PFT) initially due to poor effort. A detailed environmental history review noted exposure to birds (pigeons) for a duration of around one year, as the family were keeping them in-house. Testing for specific bird IgG to pigeon test was not available in our institute and hence this was sent to the Mayo Clinic, USA. Further tests showed complete blood count showed leukocytosis with neutrophilia, an erythrocyte sedimentation rate (ESR) of 37 mm/h (N: 0–10 mm/h), and a C-reactive protein (CRP) count of 78 mg/L (N: 0–8 mg/L). Renal function and liver enzymes were normal, A sweat chloride test showed normal results, and measures of immunoglobulins A, M, G, E, IgG subclasses, the flow cytometry lymphocytic subset, and the HIV combo test all came back normal. Autoimmune work up showed a negative antinuclear antigen screen (ANA) and anti-glomerular basement membrane antibodies. Echocardiography showed normal cardiac function and no signs of pulmonary hypertension. The child underwent a flexible bronchoscopy evaluation that showed normal airway anatomy and dynamics, with no signs of chronic airway inflammation. The bronchoalveolar lavage (BAL) sample showed unsatisfactory results for cell and differential counts, but the BAL microbiology and virus results were negative.

**Figure 3:**
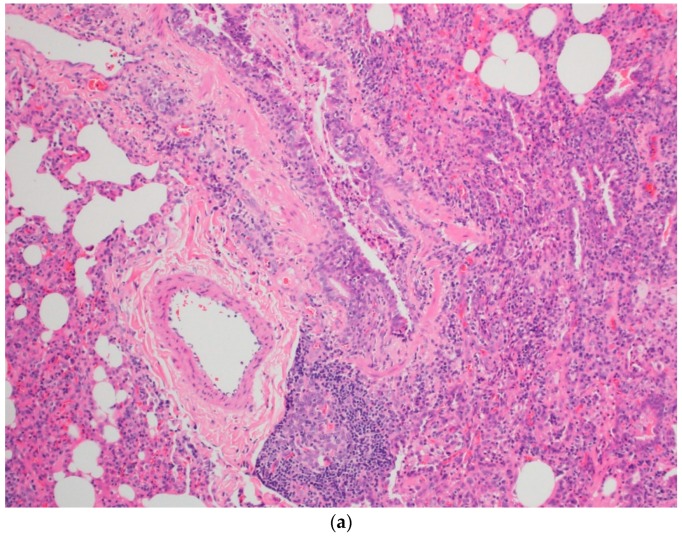

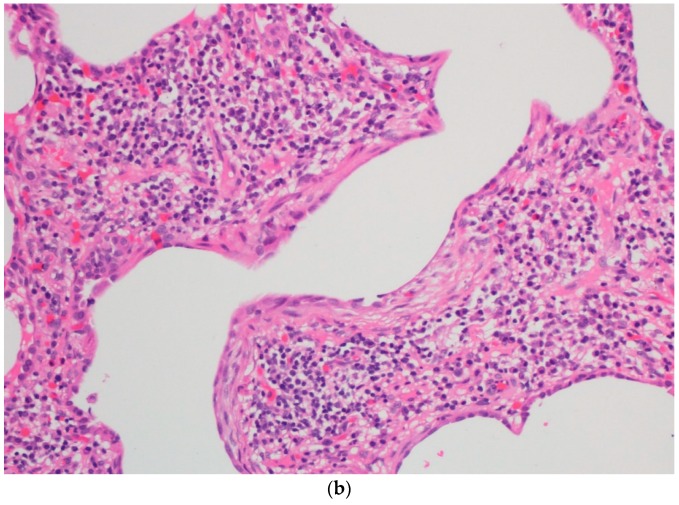
Sections show wedge lung biopsy characterized by patchy inflammatory process. The inflammation is bronchiolocentric (**a**) with significant extension into the interstitium consisting of sheets of lymphocytes, plasma cells, foamy macrophages, and rare eosinophils as well as neutrophils (**b**). No definitive granulomas, desquamation, vasculitis, significant eosinophilic infiltrate, Langerhans’ cells, or fibrosis are seen. Special stains for fungi, acid-fast organisms, and viral inclusions are negative.

The child’s symptoms continued to worsen with progressive dyspnea and dry cough, and the decision was made to undergo an open lung biopsy with histopathology findings suggestive of acute hypersensitivity pneumonitis (HP) (Figure 3a,b). All pigeons were removed from the household, and lifetime avoidance of exposure to pigeons was recommended. He was further treated with pulse steroid therapy (30 mg/kg/day) for 3 days with the plan to give 3–6 cycles of monthly pulse steroids with follow up in an outpatient clinic.

The child was seen on a monthly basis in the outpatient clinic, and his specific bird IgG to pigeon feathers came back positive (131 mcg/mL, normal <22). He showed dramatic clinical improvement after removal of all pigeons from the house and on monthly pulse steroid therapy, and he was subsequently weaned off oxygen with improvement in his lung volume in his PFT results (Table 1).

## 2. Discussion

Bird Fancier’s Lung (BFL) is a rare entity of HP in pediatrics. It occurs due to a nonatopic immunologic response to repeated or intense inhalation of avian (bird) proteins/antigens found in the feathers or droppings of many species of birds, leading to an immune-mediated inflammatory reaction in the respiratory system [1].

The pathophysiology of HP is complex and not well understood. Numerous organic and inorganic antigens can cause immune dysregulation, leading to an immune-related antigen–antibody response (IgG against the offending antigen) [2,3,4]. Others have suggested underlying genetic factors that may contribute to the development of BFL [1]. Pathologically, finding of lymphocytic plasma cell infiltration and foamy macrophages were found in our patient’s histopathological lung biopsy, which are distinguishable parenchymal changes that occur during the acute/subacute phase of an illness. No definitive granulomas were seen (Figure 3a,b) [4].

Few case reports in the pediatric age group are available in the literature. The largest publication of pediatric BFL was reported by Morell et al. in 2008 and included 86 patients, over a 30-year period, with a rate of up to 8% of children younger than 15 years [5].

HP can be classified into three disease stages—acute, subacute, and chronic—all with unique clinical presentations [5]. Clinical, radiological, and histopathologic findings depend on the disease stage at the time of evaluation. During the acute/subacute phase of HP, symptoms and clinical findings are nonspecific and often misdiagnosed as asthma, acute tuberculosis (TB) infection, or acute viral lower respiratory tract infection, which share similar clinical and radiological findings in our case report [6].

Currently, diagnosing BFL is challenging. There is uncertainty about the significant exposure duration to such antigens leading to clinical symptoms [3]. The criteria for diagnosing HP are not well standardized, and management guidelines in the pediatric age group are still lacking [5,7,8]. We believe that our patient’s superimposed viral infection (bocavirus) either triggered or flared up the disease. Our case lacked careful environmental exposure history (such as birds); the patient’s insidious presentation during the acute/subacute phase overlapped with the underlying viral illness, which made it difficult for the health care providers to make the appropriate diagnosis during the first admission. This is why the disease often goes unrecognized in the pediatric population [3].

Treatment, on the other hand, is also controversial. Environmental avoidance of the offending antigen has been reported as the sole treatment, which helps the resolution of symptoms in a few patients [3]. Others have tried oral steroids [5,8,9,10]. Another approach is treatment with intravenous pulse steroids for 3–6 months. This is considered for severe cases [3] and was recently reported in a case study poster at the latest American Thoracic Society (ATS) international conference [11]. In such cases, it is crucial not to delay the diagnosis until the child’s condition progresses to the chronic stage, with irreversible restrictive pathology due to fibrosis [12].

Upon follow up, our patient had received six doses of IV pulse steroids (30 mg/kg/day) for 3 days on a monthly basis, with the first dose received within 1 month of the onset of clinical symptoms with complete avoidance of pigeon exposure. He showed significant improvement of clinical symptoms after the second dose in terms of resolution of the tachypnea, shortness of breath and the ability to perform regular school exercise. Home oxygen therapy was successfully weaned off during outpatient follow up with improvement in his pulmonary function test results (Table 1).

## 3. Conclusions

BFL is a rare entity of HP that occurs in children. Environmental exposure history is an important factor for early disease recognition. The initiation of pulse steroid therapy in severe cases during the acute/subacute phase dramatically improves clinical symptoms and prognosis, preventing disease progression and lung fibrosis development.

## Figures and Tables

**Table 1:** Pulmonary function test results after first, third, and sixth dose of pulse steroid therapy.

Pulmonary Function Test	First Dose Therapy (Pred%)	Third Dose Therapy (Pred%)	Sixth Dose Therapy (Pred%)
FEV1	74	63	82
FVC	69	69	81
FEV1/FVC	107	91	100
DLco	51	63	69
TLC	64	60	72
RV	75	61	86
RV/TLC	111	97	112

## Abbreviations

ILD	interstitial lung disease
BFL	Bird Fancier’s Lung
HP	hypersensitivity pneumonitis
PICU	pediatric intensive care unit
HFNC	high-flow nasal cannula
PPD	purified protein derivative
ESR	erythrocyte sedimentation rate
CRP	C-reactive protein
CXR	chest X-ray
PCR	polymerase chain reaction
CT scan	computed tomography scan
TB	tuberculosis
BAL	broncho-alveolar lavage
ATS	American Thoracic Society
